# Assessment of risk factors for early-onset deep surgical site infection following primary total hip arthroplasty for osteoarthritis

**DOI:** 10.5194/jbji-6-443-2021

**Published:** 2021-12-08

**Authors:** Jonathan Bourget-Murray, Rohit Bansal, Alexandra Soroceanu, Sophie Piroozfar, Pam Railton, Kelly Johnston, Andrew Johnson, James Powell

**Affiliations:** 1 Department of Surgery, Division of Orthopaedic Surgery, University of Calgary, Calgary, Alberta, Canada; 2 Alberta Bone and Joint Health Institute, Calgary, Alberta, Canada; 3 Department of Medicine, Division of Infectious Diseases, University of Calgary, Calgary, Alberta, Canada

## Abstract

The aim of this study was to determine the incidence, annual trend, and perioperative outcomes and identify risk factors of early-onset ( ≤90 d) deep surgical site infection (SSI) following primary total hip arthroplasty (THA) for osteoarthritis. We performed a retrospective study using prospectively collected patient-level data from January 2013 to March 2020. The diagnosis of deep SSI was based on the published Centre for Disease Control/National Healthcare Safety Network (CDC/NHSN) definition. The Mann–Kendall trend test was used to detect monotonic trends. Secondary outcomes were 90 d mortality and 90 d readmission. A total of 22 685 patients underwent primary THA for osteoarthritis. A total of 46 patients had a confirmed deep SSI within 90 d of surgery representing a cumulative incidence of 0.2 %. The annual infection rate decreased over the 7-year study period ( p=0.026 ). Risk analysis was performed on 15 466 patients. Risk factors associated with early-onset deep SSI included a BMI > 30 kg m ${}^{-2}$ (odds ratio (OR) 3.42 [95 % CI 1.75–7.20]; p<0.001 ), chronic renal disease (OR, 3.52 [95 % CI 1.17–8.59]; p=0.011 ), and cardiac illness (OR, 2.47 [1.30–4.69]; p=0.005 ), as classified by the Canadian Institute for Health Information. Early-onset deep SSI was not associated with 90 d mortality ( p=0.167 ) but was associated with an increased chance of 90 d readmission ( p<0.001 ). This study establishes a reliable baseline infection rate for early-onset deep SSI after THA for osteoarthritis through the use of a robust methodological process. Several risk factors for early-onset deep SSI are potentially modifiable, and therefore targeted preoperative interventions of patients with these risk factors is encouraged.

## 1 Introduction

Total hip arthroplasty (THA) is known to be highly successful at alleviating pain and disability, providing substantial improvement in quality of life (Harris, 2009). Nationally, the number of THA procedures has increased over 20 % in the last 5 years (Canadian Institute for Health Information, 2021) and is projected to grow in step with the aging demographics of the population (Singh et al., 2019; Kurtz et al., 2007). As the demand continues to increase, so will the impact on healthcare costs, which are currently estimated to be over CAD 1.4 billion annually in Canada (Canadian Institute for Health Information, 2021). Despite improved infection prevention protocols and surgical technique, surgical site infection (SSI) and periprosthetic joint infection (PJI) following THA continue to be a serious problem. These infections result in significant individual morbidity and increase mortality risk (Gundtoft et al., 2017; Moore et al., 2015; Zmistowski et al., 2013). Due to the projected increase in demand for THA, it is highly likely that the absolute number of SSI cases will continue to increase (Kamath et al., 2015; Perfetti et al., 2017; Premkumar et al., 2021).

It is imperative to understand the most relevant risk factors associated with SSI in order to identify modifiable factors in order to improve patient care and hospital productivity and positively impact healthcare spending. Unfortunately, current large-scale studies lack comprehensiveness, which limits the generalizability of their findings and relevant outcomes. First, many do not study homogeneous groups of patients with a common primary indication for THA (i.e., osteoarthritis) but rather mixed patient groups, which does not allow for consistent comparison (Lenguerrand et al., 2018; Bozic et al., 2012). Second, variability in the definition of deep SSI can be problematic, especially when limited to patients requiring revision surgery – an indication biased by the judgment of the surgeon (Perfetti et al., 2017; Arthroplasty Collaborative Mac TM, 2020; Gundtoft et al., 2015). Doing so inherently introduces the problem of misclassification and poorly estimates the true incidence of infection. To correct this, the definition for a SSI must use validated criteria, such as those of the Centre for Disease Control/National Healthcare Safety Network (CDC/NHSN) or the Musculoskeletal Infection Society's (MSIS) (Parvizi et al., 2013; CDC/NHSN, 2021). Finally, the importance of various patient and surgical associated risk factors for SSI may vary by type of infection. For example, early-onset ( ≤90 d) infections account for approximately 14 %–25 % of all periprosthetic joint infections following THA (Lenguerrand et al., 2018; Manning et al., 2020). Early-onset deep SSI is of particular interest as it occurs shortly after surgery and is therefore more likely to be associated with risk factors present at the time of the surgical procedure – thus possibly modifiable (Zimmerli et al., 2004).

The aim of this study was to determine the incidence, annual trend, and perioperative outcomes of early-onset ( ≤90 d) deep SSI, as defined by the CDC/NHSN, following primary THA for osteoarthritis. We also wanted to identify patient and surgical risk factors specifically related to early-onset deep SSI. Secondary outcomes of interest were 90 d mortality and 90 d readmission.

## 2 Methods

### 2.1 Study design

This was a retrospective population-based cohort study using prospectively collected patient-level data extracted from the provincial administrative data repositories that are stored and maintained by the Alberta Bone and Joint Health Institute (ABJHI). The ABJHI is a non-for-profit organization that collates and maintains population-based provincial musculoskeletal data from all hospitals across Alberta (Canada), in a country with universal healthcare coverage. This study was approved by the local Conjoint Health Research Ethics Board and by the Provincial Health Services Research Department to allow access to patient-specific data.

### 2.2 Study population

The ABJHI data repositories were searched to identify all patients who underwent a primary, unilateral THA for osteoarthritis between 1 January 2013 and 1 March 2020. This was achieved using the Canadian Classification of Health Interventions (CCI) codes 1VA53LAPN and 1VA53LLPN and their variations. Patients were excluded if they had previous ipsilateral hip fracture or surgery, post-traumatic arthritis, or rheumatoid arthritis, underwent simultaneous bilateral THA, or had any admitting diagnosis other than primary osteoarthritis.

The ABJHI partners with the provincial Infection Prevention and Control (IPC) authorities for prospective surveillance and data collection of all patients admitted to any hospital facility in the province for arthroplasty (Rennert-May et al., 2016; Rusk et al., 2016). The surveillance protocol begins immediately following the surgery, continues during the acute hospital stay, and is actively performed until 90 d postoperative (Canadian Institute for Health Information, 2020). IPC surveillance is conducted by infection control specialists as part of the provincial SSI surveillance program, and all patient information is entered into a centralized web-based platform. Every patient is linked to the Discharge Abstract Database (DAD) and to the National Ambulatory Care Reporting System by the local analytics department. Cases of infection are detected by electronic review of microbiology laboratory results; review of patient charts, physician records, and pharmacy data; re-operation records; readmissions; emergency visit records; and clinic visit records, including observation of the surgical incision (Rennert-May et al., 2016). The diagnosis of a deep SSI is determined based on the criteria outlined by the CDC/NHSN (Table 1) (CDC/NHSN, 2021).

**Table 1:** CDC/NHSN definition of deep surgical site infection.

The date of event occurs within 30 or 90 d after the NHSN	
operative procedure ${}^{*}$ (where day 1 = the procedure date)	
AND	
Involves deep soft tissues of the incision (for example, fascial	
and muscle layers)	
AND	
Patient has at least one of the following:	
A. purulent discharge from incision	
B. a deep incision that spontaneously dehisces or is	
deliberately opened	
AND	
Organism(s) identified from the deep soft tissues of the	
incision by a culture- or non-culture-based microbiologic	
testing method, which is performed for purposes of	
clinical diagnosis or treatment. A culture- or non-culture-	
based test from the deep soft tissues of the incision that	
has a negative finding does not meet this criterion.	
AND	
Patient has at least *one* of the following signs or	
symptoms: fever ( >38 ${}^{\circ }$ C), localized pain, or tenderness	
C. an abscess or other evidence of infection involving the	
deep incision that is detected on gross anatomical or	
histopathologic exam or imaging test	

The term physician for the purpose of application of the NHSN SSI criteria may be interpreted to mean a surgeon, infectious disease physician, emergency physician, other physician on the case, or physician's designee (nurse practitioner or physician's assistant). ${}^{*}$ NHSN category: HPRO. Operative procedure: hip prosthesis.

Data on patient characteristics, pre-surgical risk factors (comorbidities), blood transfusions, and acute hospital length of stay (LOS) were collected from electronic medical records, operating room information systems, the DAD, and IPC surveillance data. The ABJHI's pre-surgery risk factor methodology identifies the presence of comorbidities by screening pre-admission comorbidities in DAD and in the Canadian Institute for Health Information (CIHI) Population Grouper data within 5 years prior to the arthroplasty event. CIHI Population Grouper data are extracted from provincial administrative data sources to identify the presence of 226 discrete health conditions, which are aggregated into 16 health profile categories, 13 of which were of interest for this study.

### 2.3 Statistical analysis

All statistical analyses were performed using R version 4.0.5 and R Studio (RStudio Team, 2020). All collected patient data were de-identified as per provincial de-identification standards and regulations. We summarize demographic data and outcomes using descriptive statistics. Categorical variables were analyzed using a chi-squared test and Fisher's exact test, while a t test was used to summarize continuous variables. We evaluated trends in annual rates of deep SSI between January 2013 and December 2019. We used the Mann–Kendall trend test to detect monotonic trends in annual early-onset deep SSI rates during this timeframe. Multiple logistic regression was used to analyze the effect of different patient and surgical risk factors on the risk of developing a deep SSI within 90 d from surgery. Patient comorbidities were referenced by the ABJHI, in order to foster quality improvement. Secondary outcomes were analyzed using multiple logistic regressions. These included 90 d mortality and 90 d readmission rate, with adjustments made for sex, age at the time of surgery, body mass index (BMI), pre-surgery risk factor groups (comorbidities), blood transfusion, anesthesia type, and same-day discharge. A p value less than 0.05 was deemed significant.

## 3 Results

There were 22 685 patients identified who received a primary THA for osteoarthritis between 1 January 2013 and 1 March 2020. Of these, 46 patients were diagnosed with a deep SSI within 90 d from surgery (Table 2). The cumulative incidence for early-onset deep SSI during the study period was 0.2 %. The annual rate of deep SSI was found to have significantly decreased over the 7-year study period ( p=0.026 ).

**Table 2:** Annual number of confirmed deep surgical site infection cases within 90 d of surgery between January 2013 and March 2020.

Year	No. of	No. of deep	Incidence
	surgeries	SSI cases	(%)
2013	2765	10	0.36 %
2014	2914	7	0.24 %
2015	2911	10	0.34 %
2016	3077	7	0.23 %
2017	3275	6	0.18 %
2018	3509	2	0.06 %
2019	3636	4	0.11 %
2020	598	0	0 %

Total no. of THA surgeries: 22 685; total no. of deep SSI cases: 46. We used the Mann–Kendall trend test to detect monotonic trends in annual early-onset deep SSI rates during this timeframe.

### 3.1 Risk factors for deep surgical site infection

Due to some missing patient demographic and surgery characteristic data, only 15 466 patients could be included for the risk analysis, 42 of whom developed an infection. Baseline patient and surgical characteristics investigated are summarized in Table 3.

**Table 3:** Patient demographics and surgery characteristics.

	No deep SSI	Deep SSI	p value
	( N=15424 )	( n=42 )	
Age, years (mean, SD)	65.4 (11.2)	69.0 (10.5)	0.032
Sex, male ( n , %)	6826 (44.3 %)	17 ( 40.5 %)	0.736
BMI, kg m ${}^{-2}$ (mean)			
<30	8394 (54.4 %)	11 (26.2 %)	<0.01
≥30	7030 (45.6 %)	31 (73.8 %)	
Comorbidities ${}^{*}$			
Asthma	948 (6.1 %)	2 (4.8 %)	0.959
Cancer	2221 (14.4 %)	10 (23.8 %)	0.130
Cardiac illness	3641 (23.6 %)	21 (50.0 %)	<0.001
Chronic hepatic conditions	195 (1.3 %)	1 (2.4 %)	1.000
Chronic pulmonary conditions	1556 (10.1 %)	9 (21.4 %)	0.029
Chronic renal conditions	375 (2.4 %)	5 (11.9 %)	<0.001
Depression	2,359 (15.3 %)	11 (26.2 %)	0.081
Dementia	143 (0.9 %)	1 (2.4 %)	0.861
Diabetes	2,072 (13.4 %)	9 (21.4 %)	0.197
Drug and/or alcohol abuse	742 (4.8 %)	2 (4.8 %)	1.000
History of DVT or PE	510 (3.3 %)	2 (4.8 %)	0.925
Stroke	201 (1.3 %)	1 (2.4 %)	1.000
Moderate or severe mental health	505 (3.3 %)	3 (7.1 %)	0.331
Perioperative characteristics			
ASA score ( n )			
≤2	12 137 (78.7 %)	26 (61.9 %)	0.014
≥3	3287 (21.3 %)	16 (38.1 %)	
Blood transfusion	754 (4.9 %)	4 (9.5 %)	0.302
Anesthetic			
General	2535 (16.4 %)	3 (7.1 %)	0.254
Spinal	12 054 (78.2 %)	36 (85.7 %)	
Combined	835 (5.4 %)	3 (7.1 %)	
Surgical time			
<90 min	5694 (36.9 %)	20 (47.6 %)	0.353
90–119 min	6024 (39.1 %)	14 (33.3%)	
≥120 min	3706 (24 %)	8 (19 %)	
Same-day discharge	549 (3.6 %)	0 (0 %)	0.408
Surgeon volume ( <30 THA per year)	1817 (11.8 %)	7 (16.7 %)	0.459
Length of hospital stay, days (SD)	3.30 (2.91)	4.67 (3.27)	0.009

Fisher's exact test. ${}^{*}$ Comorbidities were captured using health conditions classified in the CIHI Population Risk Grouper data. “Cardiac conditions” include acute myocardial infarction or arrest, arrhythmia, coronary artery disease, cardiac valve disease, malformation of cardiovascular system, heart failure. “Chronic hepatic conditions” include chronic liver disease, including hepatic cirrhosis. “Chronic pulmonary conditions” include congenital disorder of the respiratory system, chronic obstructive pulmonary disease, pulmonary hypertension, respiratory failure, cystic fibrosis, tuberculosis disease, and other chronic lung disease. “Chronic renal conditions” include chronic kidney disease/failure. “Moderate or severe mental health” includes delusional disorder (inc. schizophrenia), bipolar/manic mood disorder, eating disorder, intellectual disorder/delay, and mental disorder resulting from brain injury or other illness. DVT, deep vein thrombosis; PE, pulmonary embolism.

On multiple logistic regression analysis, elevated BMI > 30 kg m ${}^{-2}$ (odds ratio (OR) 3.42 [95 % CI] 1.75 to 7.20]; p<0.001 ), chronic renal disease (OR, 3.52 [95 % CI 1.17 to 8.59]; p=0.011 ), and cardiac illness (OR, 2.47 [95 % CI 1.30 to 4.69]; p=0.005 ) were associated with increased risk of developing early-onset deep SSI following primary THA (Table 4).

**Table 4:** Risk factors for early-onset deep surgical site infection.

Risk factor	Odds ratio [ 95 % CI ]	p value
BMI ( ≥30 kg m ${}^{-2}$ )	3.42 [ 1.75–7.20 ]	0.001
Cardiac illness	2.47 [ 1.30–4.69 ]	0.005
Chronic renal conditions	3.52 [ 1.17–8.59 ]	0.011

Multiple logistic regression. Health conditions are as classified in the CIHI Population Risk Grouper data. “Cardiac conditions” include acute myocardial infarction or arrest, arrhythmia, coronary artery disease, cardiac valve disease, malformation of cardiovascular system, and heart failure. “Chronic renal conditions” include chronic kidney disease/failure.

### 3.2 Perioperative outcomes

Adjusted secondary outcomes are shown in Table 5. These data were adjusted by age, sex, BMI comorbidities, blood transfusion, anesthesia type, and same-day discharge using multiple logistic regression to determine the 90 d odds ratio for readmission and mortality. Developing an infection within 90 d of surgery was associated with increased odds of requiring readmission within 90 d from surgery (OR, 65.90 [95 % CI 33.73–130.96]; p<0.001 ) but not associated with 90 d mortality (OR 4.63 [95 % CI 0.45–23.02]; p=0.167 ).

**Table 5:** Adjusted secondary outcomes.

Outcomes	Odds ratio [ 95 % CI ]	p value
	(infection vs. no infection)	
90 d readmission	65.90 [ 33.73–130.96 ]	<0.001
90 d mortality	4.63 [ 0.45–23.02 ]	0.167

Adjusted by age, sex, BMI, comorbidities, and blood transfusion using multiple logistic regression.

## 4 Discussion

This study aimed to determine the true incidence of early-onset deep SSI following primary THA for osteoarthritis using prospectively collected population-based data extracted from our provincial administrative data repositories. The findings from this study do however only reflect data from patients with early-onset deep SSI and not those of patients who developed an acute hematogenous or chronic periprosthetic joint infection years following surgery. In fact, to the best of our knowledge, early-onset ( ≤90 d) SSI only accounts for approximately 14 % to 25 % of all periprosthetic joint injections (Lenguerrand et al., 2018; Manning et al., 2020). However, the aim of this study was to identify associated risk factors that are much more likely to influence outcomes at the time of the index surgery. By using data collected systematically by IPC, who perform in-depth case reviews and use validated criteria to diagnose infection (CDC/NHSN), we present outcomes and identify relevant risk factors while minimizing interobserver variability.

We identified a total of 22 685 patients who underwent a THA for primary osteoarthritis between 1 January 2013 and March 2020. A total of 46 patients had a confirmed diagnosis of a deep SSI within 90 d of surgery, representing an incidence of 0.2 %. This incidence is in keeping with other large cohort studies (Arthroplasty Collaborative Mac TM, 2020; RStudio Team, 2020; Ong et al., 2009). Lenguerrand et al. (2017) analyzed 623 253 primary hip procedures with varying admitting diagnosis and reported an incidence of early-onset deep SSI of 0.4 % (Lenguerrand et al., 2017), while Mahomed et al. (2003) analyzed United States Medicare data and reported an incidence of 0.24 % for early-deep SSI ( ≤90 d) (Mahomed et al., 2003). We believe our lower incidence compared to the study by Lenguerrand et al. (2017) may be explained by our study's inclusion criteria – specific to patients with primary osteoarthritis – and therefore do not include presumably higher risk populations (i.e., rheumatoid arthritis). There is only one other publication to report on deep SSI after THA for primary osteoarthritis, citing a cumulative incidence of 0.48 % at 1 year and rising to 1.44 % at 15 years, but no estimate of early-onset deep SSI (Arthroplasty Collaborative Mac TM, 2020).

Our study found that the annual rate of early-onset deep SSI following primary THA for osteoarthritis decreased between January 2013 and December 2019. This may have been secondary to ongoing changes in the arthroplasty care pathways in our province featuring central intake clinics, dedicated inpatient and operating room resources, routine use of tranexamic acid and transfusion protocols, surgical preparation and medical optimization in the form of comprehensive preoperative assessment, and mobilization on the day of surgery (Gooch et al., 2009). Of note, decolonization of patients preoperatively is not routinely performed across our province. Many of these implementations are more likely to have an immediate impact on early perioperative outcomes, including a decreased risk of developing an infection within 90 d from surgery. However, these findings oppose those from other relevant studies (Lenguerrand et al., 2017; Kurtz et al., 2012; Dale et al., 2012). An observational study using data from the United States National Inpatient Sample showed the incidence of PJI in patients having THA increased from 1.99 % to 2.18 % between 1999 and 2009 (Kurtz et al., 2012). Such findings are corroborated in the Nordic Arthroplasty Registry (0.46 % to 0.71 % between 1999 and 2009) (Dale et al., 2012). In addition, Lenguerrand et al. (2017) reviewed THAs performed in England and Wales from the National Joint Registry and showed that the prevalence of revision due to PJI in the 90 d following primary THA had risen 2.3-fold (95 % CI, 1.3 to 4.1) between 2005 and 2013 (Lenguerrand et al., 2017). Despite improvements in the arthroplasty care pathways in our province, the contrasting trend in annual prevalence between studies if likely multifactorial. However, the results in this study must be interpreted in light of the fact that any patients with any admitting diagnosis other than primary osteoarthritis were excluded. The risk of developing a SSI is known to vary according to the indication for the primary procedure. Specifically, patients operated on for primary osteoarthritis of the hip have been found to be at lower risk of being revised for PJI than those without osteoarthritis (Lenguerrand et al., 2018).

By defining cases of infection using validated criteria (CDC/NHSN), we believe the results of this study closely represent the true incidence of early-onset deep SSI in our patient population. Previous studies that defined cases of infection as those requiring a revision surgery may misrepresent the true incidence (Arthroplasty Collaborative Mac TM, 2020; Gundtoft et al., 2015; Lenguerrand et al., 2017). The reason for a revision surgery is multifactorial and at the discretion of the surgeon. Inevitably some patients are managed non-operatively with antibiotic therapy and are not captured in the data; although not a routinely acceptable treatment option, the general health of the patient must be taken into consideration. The ABJHI has created a standardized approach to improve detection and case-finding consistency which allows for improved data quality and reporting of deep SSI following total joint arthroplasty (Rennert-May et al., 2016; Rusk et al., 2016). The administrative data-triggered medical chart review with the use of IPC confirmation protocol has been shown to have a sensitivity of 90 % and specificity of 99 % (Gooch et al., 2009). This has resulted in a 1.1- to 1.7-fold increase in SSI rate compared with traditional surveillance (Rusk et al., 2016).

Several risk factors were found to be associated with the development of early-onset deep SSI: a BMI > 30 kg m ${}^{-2}$ , chronic renal disease, and cardiac illness. Interestingly, risk factors known to be important for late-onset deep SSI such as diabetes, drug and/or alcohol abuse, longer operative time, and blood transfusion were not found to be significant risk factors in this study (Arthroplasty Collaborative Mac TM, 2020; Ong et al., 2009; Kunutsor et al., 2016; Tan et al., 2018). Although only speculative, it may be that the risk factors important for early-onset deep SSI differ from those of late-onset deep SSI or that these risk factors exert different effects at different time points and that these effects may be cumulative over time. For instance, when considering the risk of diabetes on cumulative risk of PJI over time vs. no diabetes, the risk curves do not immediately diverge (Arthroplasty Collaborative Mac TM, 2020). This study may have been underpowered to detect the early effects of diabetes that might have been more obvious if we looked at later time points. In addition, it is unclear if limiting inclusion criteria to only patients with admitting diagnosis of primary osteoarthritis influenced the findings of this study.

The cause for early-onset deep SSI is likely to be multifactorial, and this study identified three potentially modifiable comorbidities. The comorbidities found to increase the risk of early-onset deep SSI following THA include an elevated BMI ( ≥30 kg m ${}^{-2}$ ), chronic renal conditions, and cardiac illness. These comorbidities can potentially be optimized before surgery during routine preoperative counselling and physical examination. Targeted preoperative investigations should be done in conjunction with internal medicine specialists or anesthetists, and safety precautions in connection with the operation are required when appropriate.

A unique feature of this study is the homogeneous patient population who received a primary THA specifically for osteoarthritis of the hip. This is important as rates of deep SSI are known to vary according to the indication for surgery. The evidence-based, standardized criteria and rigorous methodology by which early-onset deep SSI was identified allows for a high degree of confidence in the reported incidence. Lastly, this study focused exclusively on the acute postoperative period, ( ≤90 d) thus identifying risk factors more likely attributable to (but certainly not limited to) the primary surgical intervention.

The limitations of our study are those inherent to large administrative databases. Inevitably, as with any database, some variables are not collected, and therefore we could only include in our analysis variables available to the ABJHI. Accordingly, the IPC surveillance in Alberta is conducted by infection control specialists, and the diagnosis of a deep SSI is determined based on the criteria outlined by the CDC/NHSN (CDC/NHSN, 2021) and not MSIS criteria (Parvizi et al., 2013). The surveillance protocol begins immediately following the surgery (as inpatients) and is actively performed for 90 d postoperative (Canadian Institute for Health Information, 2020). While some authorities state that any infection within 90 d of the index arthroplasty should be considered acute (Parvizi and Gehrke, 2014), early SSI has also been defined as infections occurring within 3 to 4 weeks (Chotanaphuti et al., 2019). Therefore, the results from this study should be understood in light of this. In 2013, an IPC data quality working group began completing an additional review of provincial admission and disease administrative codes to ensure no cases of infection after total joint arthroplasty were being missed across the province (Rennert-May et al., 2016). This process allowed us, for the purpose of this study, to determine, with high accuracy, the true annual incidence of early-onset deep SSI across the province and to quantify whether the annual rate of deep SSI had changed between 2013 and 2019. Secondly, despite the large number of included patients, 7219 (31.8 %) patients were omitted from the risk analysis. These patients were excluded due to missing data or issues involving data completeness – a limitation for studies using administrative databases that is well described in the literature (Smith et al., 2018). Lastly, while the aim of this study was to identify risk factors present at the time of the index surgery, the authors acknowledge that early-onset SSI may be related to more virulent organisms which manifest at an earlier time point. In light of this, less virulent pathogens may present later despite gaining entry to the surgical site at the time of the operation. Despite these shortcomings, the ABJHI database is one of the largest institutes that prospectively collects and analyzes data for measuring outcomes against international benchmarks using carefully selected data elements and key performance indicators.

## 5 Conclusion

With the rise in THA, an increasing number of patients are at risk of developing a deep SSI involving the joint and adjacent deep tissues. This study reports a rate of deep surgical site infection of 0.2 % in a cohort of patients treated surgically for osteoarthritis of the hip at the 90 d review mark using CDC criteria for diagnosis. This work establishes a reliable population-based baseline infection rate for early-onset deep SSI after THA for osteoarthritis. It also provides valuable insight into risk factors associated with developing an early-onset deep SSI. Although the absolute incidence of deep SSI is low, growing demand for arthroplasty will have substantial implications for service delivery and healthcare costs. In order to successfully devise strategies and interventions that can reduce infection rates and improve outcomes, we must identify patients with modifiable risk factors to counsel and optimize prior to surgery. Use of targeted interventions to influence these risk factors could be effective in reducing the incidence of infection in the future.

## Data Availability

Data are owned by the Alberta Bone and Joint Health Institute (ABJHI) and cannot be provided without permission by the local Conjoint Health Research Ethics Board and by the Provincial Health Services Research Department to allow access to patient-specific data.
