# *Corrigendum to* “Assessment of risk factors for early-onset deep surgical site infection following primary total hip arthroplasty for osteoarthritis” published in J. Bone Joint Infect., 6, 443–450, 2021

**DOI:** 10.5194/jbji-7-151-2022

**Published:** 2022-07-11

**Authors:** Jonathan Bourget-Murray, Rohit Bansal, Alexandra Soroceanu, Sophie Piroozfar, Pam Railton, Kelly Johnston, Andrew Johnson, James Powell

**Affiliations:** 1 Department of Surgery, Division of Orthopaedic Surgery, University of Calgary, Calgary, Alberta, Canada; 2 Alberta Bone and Joint Health Institute, Calgary, Alberta, Canada; 3 Department of Medicine, Division of Infectious Diseases, University of Calgary, Calgary, Alberta, Canada

The authors regret to report a mistake that has led to important errors in our original article (Bourget-Murray et al., 2021). The discrepancies in the results of the original article included Tables 2, 3, and 4. The correct methodology and results, including incidence, annual trend, perioperative outcomes, and risk factors of early-onset ( ≤90 d) deep surgical site infection (SSI) following primary THA for osteoarthritis in our study population, can be found below.

## 2 Methods

Deep SSIs were diagnosed by following the criteria outlined by the CDC/NHSN (CDC/NHSN, 2021). The ABJHI categorizes deep SSI by grouping patents who satisfy the CDC/NHSN definition of “deep surgical site infection” or “organ/space infection” together as both are a reflection of periprosthetic hip infections that extend deep to the fascia and involve the deep soft tissues and joint and therefore, from a surgical point of view, are treated in similar fashion.

## 3 Results

There were 26 943 patients identified who received a primary THA for osteoarthritis between 1 January 2013 and 1 March 2020. Of these, 328 patients were diagnosed with a deep SSI within 90 d from surgery (Table 2). The cumulative incidence for early-onset deep SSI during the study period was 1.2 %. The annual rate of early-onset deep SSI was not found to have significantly decreased over the 7-year study period ( p=0.29 ).

**Table 2:** Annual number of confirmed complex surgical site infection cases within 90 d of surgery between January 2013 and March 2020.

Year	Total no.	No. of complex	Incidence
	of surgeries	SSI	
2013	3346	53	1.6 %
2014	3485	45	1.3 %
2015	3523	35	1.0 %
2016	3694	55	1.5 %
2017	3902	39	1.0 %
2018	4130	49	1.2 %
2019	4180	47	1.1 %
2020	683	5	0.7 %

Total THA surgeries: 26 943; total deep SSI: 328. We used the Mann–Kendall trend test to detect monotonic trends in annual early-onset deep SSI rates during this timeframe.

**Table 3:** Patient demographics and surgery characteristics.

		No infection ( N=18124 )	Deep SSI ( n=257 )	P value
Age, mean years (SD)		65.5 (11.2)	66.9 (11.3)	0.055
Sex, n male (%)		8253 (45.5 %)	132 (51.4 %)	0.072
BMI, kg m ${}^{-2}$ (mean)				
	<30	9582 (52.9 %)	68 (26.5 %)	<0.001
	≥30	8542 (47.1 %)	189 (73.5 %)	
Co-morbidities*				
Asthma		1202 (6.6 %)	31 (12.1 %)	<0.001
Cancer		2683 (14.8 %)	28 (10.9 %)	0.096
Cardiac illness		4485 (24.7 %)	97 (37.7 %)	<0.001
Chronic hepatic conditions		252 (1.4 %)	7 (2.7 %)	0.125
Chronic pulmonary conditions		1976 (10.9 %)	59 (23.0 %)	<0.001
Chronic renal conditions		525 (2.9 %)	20 (7.8 %)	<0.001
Depression		2837 (15.7 %)	59 (23.0 %)	0.002
Dementia		181 (1.0 %)	3 (1.2 %)	1
Diabetes		2610 (14.4 %)	54 (21.0 %)	0.004
Drug and/or alcohol abuse		975 (5.4 %)	29 (11.3 %)	<0.001
Deep vein thrombosis		658 (3.6 %)	20 (7.8 %)	<0.001
Human immunodeficiency virus		13 (0.1 %)	1 (0.4 %)	0.488
Stroke		247 (1.4 %)	3 (1.2 %)	1
Moderate or severe mental health		643 (3.5 %)	20 (7.8 %)	<0.001
Peri-operative characteristics				
ASA score ( n )				
	≤2	13 924 (76.8 %)	161 (62.6 %)	<0.001
	≥3	4200 (23.2 %)	96 (37.4 %)	
Blood transfusion		951 (5.2 %)	30 (11.7 %)	<0.001
Anesthetic				
	General	2966 (16.4 %)	37 (14.4 %)	0.011
	Spinal	14 165 (78.2 %)	195 (75.9 %)	
	Combined	993 (5.5 %)	25 (9.7 %)	
Surgical time				
	<90 min	6760 (37.3 %)	104 (40.5 %)	0.553
	90–119 min	7062 (39.0 %)	97 (37.7 %)	
	≥120 min	4302 (23.7 %)	56 (21.8 %)	
Same day discharge		605 (3.3 %)	2 (0.8 %)	0.035
Surgeon volume ( <30 THA yr ${}^{-1}$ )		2229 (12.3 %)	45 (17.5 %)	0.015
Hospital volume, per year/per hospital, mean (SD)		60.8 (39.4)	68.0 (40.6)	0.005
Length of hospital stay, days (SD)		3.38 (3.18)	5.46 (7.18)	<0.001

Fisher's exact test. * Co-morbidities were captured using health conditions classified in The CIHI Population Risk Grouper data: *Cardiac conditions* include acute myocardial infarction or arrest, arrhythmia, coronary artery disease, cardiac valve disease, malformation of cardiovascular system, heart failure. *Chronic hepatic conditions* include chronic liver disease including hepatic cirrhosis. *Chronic pulmonary conditions* include congenital disorder of the respiratory system, chronic obstructive pulmonary disease, pulmonary hypertension, respiratory failure, cystic fibrosis, tuberculosis disease and other chronic lung disease. *Chronic renal conditions* include chronic kidney disease/failure. *Moderate or severe mental health* includes delusional disorder (incl. schizophrenia), bipolar/manic mood disorder, eating disorder, intellectual disorder/delay and mental disorder resulting from brain injury or other illness. DVT, deep vein thrombosis; PE, pulmonary embolism.

**Table 4:** Risk factors for early-onset periprosthetic joint infection.

Risk factor	Odds ratio	p value
	[95 % CI]	
Sex, male	1.23 [0.95–1.58]	0.111
BMI ( ≥30 kg m ${}^{-2}$ )	3.16 [2.39–4.22]	<0.001
Cardiac conditions	1.30 [0.99–1.71]	0.056
Chronic pulmonary conditions	1.70 [1.23–2.31]	0.001
Chronic renal conditions	1.62 [0.95–2.61]	0.059
Drug and/or alcohol abuse	1.77 [1.15–2.65]	0.007
Deep vein thrombosis	1.64 [0.98–2.58]	0.043
Moderate or severe mental health	1.58 [0.94–2.52]	0.069
Blood transfusion	1.89 [1.22–2.84]	0.003
Same day discharge	0.40 [0.07–1.26]	0.197
Surgeon volume ( <30 THA yr ${}^{-1}$ )	1.41 [1.00–1.94]	0.042
Hospital volume (per year/per hospital)	1.45 [1.06–1.99]	0.020
Acute length of stay	1.04 [1.02–1.05]	<0.001
Multiple logistic regression.		

### 3.1 Risk factors for deep surgical site infection

Due to some missing patient demographic and surgery characteristic data, only 18 381 patients could be included for analysis, 257 of whom developed a deep SSI. Baseline patient and surgical characteristics investigated are summarized in Table 3. Multiple logistic regression analysis revealed BMI ≥30 kg m ${}^{-2}$ (OR, 3.16 [95 % CI, 2.39 to 4.22]; p<0.001 ), chronic pulmonary disease (OR, 1.70 [95 % CI, 1.23 to 2.31]; p=0.001 ), drug and/or alcohol abuse (OR, 1.77 [95 % CI, 1.15 to 2.65]; p=0.007 ), deep vein thrombosis (OR, 1.64 [95 % CI, 0.98 to 2.58]; p=0.043 ), blood transfusion (OR, 1.89 [95 % CI, 1.22 to 2.84]; p=0.003 ), surgeon volume ( <30 THA yr ${}^{-1}$ ; OR, 1.41 [95 % CI, 1.00 to 1.94]; p=0.042 ), hospital volume (OR, 1.45 [p95 % CI, 1.06 to 1.99]; p=0.020 ), and acute hospital LOS (OR, 1.04 [95 % CI, 1.02 to 1.05]; p<0.001 ) were associated with increased risk of developing early-onset SSI following primary THA. The complete results from regression model are presented in Table 4.

### 3.2 Perioperative outcomes

Secondary outcomes were adjusted by age, sex, BMI ( ≥30 kg m ${}^{-2}$ ), pre-surgery risk factor groups, anesthesia type, blood transfusion, same day discharged, acute LOS, surgeon volume, and hospital volume using multiple logistic regression. Developing a deep SSI within 90 d of surgery was associated with readmission within 90 d from surgery (OR, 19.43 [95 % CI, 14.76 to 25.54]; p<0.001 ) and associated with 90 d mortality (OR 7.24 [95 % CI, 2.45 to 17.21]; p<0.001 ).

## 4 Discussion

This incidence of early-onset deep SSI is higher than we previously reported (Bourget-Murray et al., 2021). In addition, we report a significantly higher incidence of periprosthetic hip infection following THA for primary osteoarthritis compared to another recent Canadian publication from Ontario which showed a cumulative incidence of 0.48 % at 1 year and rising to 1.44 % at 15 years, but no estimate of early-onset deep SSIs (Arthroplasty Collaborative Mac™, 2020) However, both studies did not identify any change in annual rate of infection during the study period. Perhaps, our higher incidence is a reflection of the active surveillance across Alberta by IPC authorities which is performed until 90 d post-operatively (Canadian Institute for Health Information, 2020). This work establishes a reliable population-based baseline infection rate for early-onset deep SSI after THA for osteoarthritis.
